# The Dynamic Nature of CO Adlayers on Pt(111) Electrodes

**DOI:** 10.1002/anie.201913412

**Published:** 2020-02-18

**Authors:** Jie Wei, Reihaneh Amirbeigiarab, Yan‐Xia Chen, Sung Sakong, Axel Gross, Olaf M. Magnussen

**Affiliations:** ^1^ Institute of Experimental and Applied Physics Kiel University 24098 Kiel Germany; ^2^ Hefei National Laboratory for Physical Sciences at Microscale University of Science and Technology of China Hefei 230026 China; ^3^ Institute of Theoretical Chemistry Ulm University Albert-Einstein-Allee 11 89081 Ulm Germany

**Keywords:** CO adlayer, density functional theory, scanning tunneling microscopy, Pt(111), surface mobility

## Abstract

CO adlayers on Pt(111) electrode surfaces are an important electrochemical system and of great relevance to electrocatalysis. The potential‐dependent structure and dynamics of these adlayers are complex and still controversial, especially in the CO pre‐oxidation regime. We here employ in situ high‐speed scanning tunneling microscopy for studying the surface phase behavior in CO‐saturated 0.1 m H_2_SO_4_ on the millisecond time scale. At potentials near the onset of CO pre‐oxidation local fluctuations in the (2×2)‐CO adlayer are observed, which increase towards more positive potentials. Above 0.20 V (vs. Ag/AgCl), this leads to an adlayer where CO_ad_ apparently reside on every top site, but still exhibit a (2×2) superstructure modulation. We interpret this observation as a dynamic effect, caused by a small number of highly mobile point defects in the (2×2)‐CO adlayer. As shown by density functional theory calculations, the CO lattice near such defects relaxes into a local (1×1) arrangement, which can rapidly propagate across the surface. This scenario, where a static (2×2) CO_ad_ sublattice coexists with a highly dynamic sublattice of partially occupied top sites, explains the pronounced CO_ad_ surface mobility during electrooxidation.

## Graphical abstract

**Adsorbed CO** on Pt(111) electrodes can become highly mobile at the onset of pre‐oxidation, despite a high adsorbate surface density. Origin of this behavior is the formation of defects in the adlayer, which enable density fluctuations and by this rapid quasi‐collective CO transport.

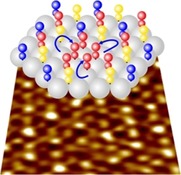

The interaction of CO with metal surfaces is a major topic in electrocatalysis as well as in gas phase heterogeneous catalysis since CO is an important intermediate or poison in many catalytic reactions.^1^ The dynamics and arrangements of CO adsorbates served as a model system in surface science studies and have been investigated widely by scanning tunneling microscopy (STM).^2^ In electrochemical environment, CO adlayers on Pt(111) are arguably the most extensively studied model system for electrochemical CO adsorption and constitute a key example for adsorbed molecular species on electrode surfaces. A wide range of both experimental and computational techniques have been applied to fundamental studies of CO on Pt(111) over the past decades.^3^

In situ studies by STM,^4^ infrared spectroscopy,^5^ sum frequency generation (SFG),^6^ as well as density functional theory (DFT) calculations^7^ have found a significant potential dependence of the CO_ad_ adsorption sites and the resulting CO adlayer structure on Pt(111) electrodes. In acidic solutions at potentials near the onset of hydrogen evolution, a hexagonal close‐packed (2×2)‐3CO adlayer is observed on Pt(111) at saturation coverage (0.75 ML). In the unit cell of this structure one CO_ad_ occupies a top site and the two other CO_ad_ neighboring three‐fold hollow sites, resulting in equidistant CO_ad_—CO_ad_ spacings of 2d_Pt_/$\sqrt{3}$ =3.2 Å (with *d*_Pt_=2.78 Å being the spacing between neighboring Pt surface atoms). Upon increasing the potential to values near the onset of CO electrooxidation, the close‐packed (2×2)‐3CO adlayer transforms into a $\left(\sqrt{19}\times \sqrt{19}\right)$ R23.4°‐13CO structure with a lower coverage of 0.68 ML and the appearance of CO_ad_ on bridge sites at the expense of CO_ad_ on three‐fold sites as well as an increase in the coverage of CO_ad_ on top sites.6a, 8 This surface phase transition was linked to CO_ad_ pre‐oxidation, that is, the onset of CO bulk oxidation at low overpotentials in a range, where a pre‐peak is observed for CO_ad_ stripping in CO‐free electrolyte.^4^, 6a, 9 The pre‐oxidation process is known to depend in a complex way on the electrode defect structure and CO_ad_ adlayer preparation conditions and its exact nature and dynamics remain under debate despite extensive studies.^6^, ^9^, ^10^ In the transition regime between (2×2)‐3CO and $\left(\sqrt{19}\times \sqrt{19}\right)$ R23.4° phase, Jung et al. reported in situ STM observations of additional CO_ad_ adlayer structures, specifically a (2×2)‐4CO and a (1×1)‐CO adlayer.^4^ These were assigned to adlayers with a coverage of 1 ML where all CO_ad_ occupy atop or near‐atop sites, corresponding to high repulsive CO_ad_—CO_ad_ interactions. The presence of such phases is highly surprising as it would imply that the CO_ad_ surface coverage (i) can be much higher than reported in all previous electrochemical or gas phase studies and (ii) transiently increases during pre‐oxidation, contrary to the expected coverage decrease.

Similar controversy exists regarding the CO_ad_ adlayer dynamics. For CO_ad_ surface diffusion on clean Pt(111) under UHV conditions diffusion coefficients of 10^−9^ cm^2^ s^−1^ were obtained at room temperature, corresponding to hopping rates between neighbor sites of ≥10^5^ s^−1^.^11^ The diffusion rate was found to increase with increasing CO_ad_ coverage (up to 10^7^ s^−1^ at saturation coverage), which was attributed to the increasing intermolecular interactions.11a For the case of CO_ad_ on Pt in electrochemical environment, where the diffusion rates were largely estimated from electrochemical kinetic data^12^ or obtained under open circuit conditions on Pt nanoparticles,^13^ the situation is less clear. In most measurements 2 to 4 orders of magnitude lower diffusion coefficients were reported.^14^ However, some studies found that the kinetics of CO oxidation could be well described by a Langmuir‐Hinshelwood mechanism using the mean‐field approximation, which indicates fast CO_ad_ surface diffusion.10b, 15

Here, we address the structure and dynamics of the CO_ad_ adlayer on Pt(111) in the pre‐oxidation regime by in situ high‐speed scanning tunneling microscopy (video‐STM),^16^ which enables direct atomic‐scale studies on millisecond time scales. Our observations are in accordance with the results of previous structural studies,^4^ but suggest a much more dynamic picture. According to the video‐STM results, the CO_ad_ mobility within the adlayer strongly increases towards more positive potentials, although the (2×2) long‐range order is maintained. We will show that a sufficient density of highly mobile point defects, generated by a slight removal of CO at potentials for CO pre‐oxidation, can rationalize this behavior. This scenario is supported by DFT calculations and conceptually similar to the “door‐opening” mechanism introduced recently to explain surface diffusion on crowded surfaces.2d, 17

The in situ video‐STM studies were performed on Pt(111) single crystal electrodes in CO‐saturated 0.1 m H_2_SO_4_ solution. At the initial potential of −0.10 V versus Ag/AgCl (KCl sat.), the molecular resolution images are in good agreement with the well‐known (2×2)‐3CO structure with a coverage of 0.75 ML (Figure 1 a). Within the unit cell one bright and two weaker maxima are visible, which are commonly identified with CO_ad_ in top and three‐fold hollow sites, respectively.6a, 8a, 9 This structure is highly stable, that is, does not exhibit significant structural changes in subsequent STM images of the videos. In the following, the adsorbates in top sites, which are more strongly bound than the three‐fold bound CO_ad_,5d will be chosen as the unit cell origin and accordingly will be called CO_ad_(0).

**Figure 1:**
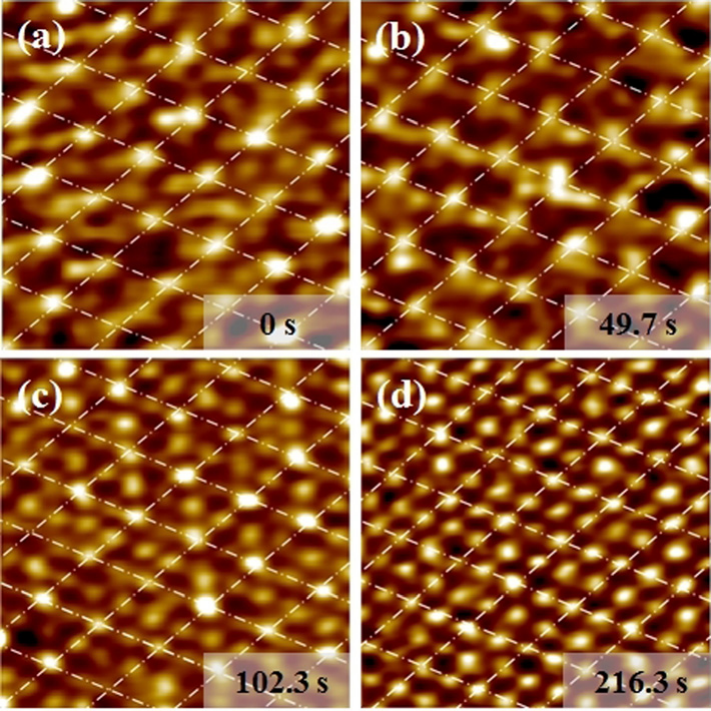
Potential‐dependent structure of the CO adlayer on Pt(111) in CO‐saturated 0.1 m H_2_SO_4_ solution. The STM images (3 nm×3 nm) were taken from a video sequence (recorded at 10 images s^−1^), in which the potential was continuously increased, and were recorded at a) −0.10 V, b) 0.12 V, c) 0.20 V, and d) 0.30 V. Gradual deviations from the (2×2) symmetry (indicated by grid of white lines) are visible with increasing potential, resulting in a modulated (1×1) structure at the most positive potentials.

Upon gradually increasing the potential into the CO pre‐oxidation region, the (2×2) periodicity of the adlayer remains largely intact. However, the distributions of the maxima inside the (2×2) unit cell, which we associate with the positions of the CO_ad_, deviates from that in the (2×2)‐3CO structure. At 0.12 V, which is close to the onset of pre‐oxidation,4b, 5d most of the prominent CO_ad_(0) of the original adlayer maintain their positions (Figure 1 b). In contrast, the more weakly‐bound CO_ad_ in the three‐fold sites of the (2×2)‐3CO structure often appear to be shifted to adjacent top, near‐top, or bridge sites. This arrangement vaguely resembles the (2×2)‐3CO‐β structure reported by Jung et al., who proposed a static shift of the two three‐fold CO_ad_ to neighboring top sites,^4^ but is more disordered. Specifically, the video‐STM images indicate that the CO_ad_ are not uniformly located at the same top sites; rather they stochastically occupy a variety of sites. Furthermore, while the lattice formed by the CO_ad_(0) is rather static, the remaining CO_ad_ exhibit rapid positional fluctuations in video sequences, recorded at a constant potential of 0.12 V. As illustrated in the sequence shown in Figure 2 a, the positions of those CO_ad_ frequently change between subsequent images (examples marked by red circles). Often, these changes are found in the same location over a sequence of several images, indicating hopping of the adsorbates between different sites on the 100 ms timescale. On the other hand, the observability of these species in the images indicates that their residence times are in the range of ≥1 ms.

**Figure 2:**
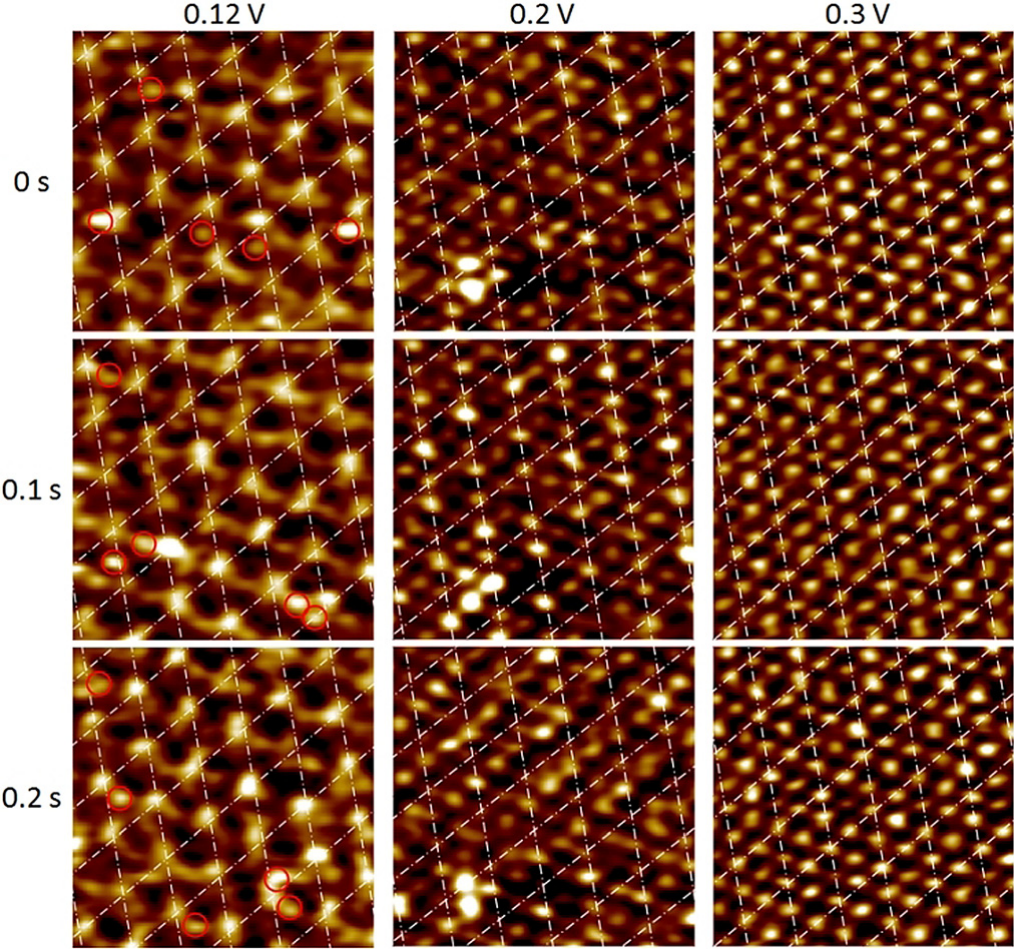
Subsequent images (3 nm×3 nm) of the CO adlayer on Pt(111) in CO‐saturated 0.1 m H_2_SO_4_ solution taken from in situ video‐STM sequences at various potentials.

Further increase of the potential to 0.20 V results again in pronounced changes in the appearance of the adlayer. Now, a rather regular hexagonal adlattice with a lattice spacing of approximately 2.78 Å and the same orientation as the (2×2)‐3CO lattice is observed (Figure 1 c). This CO adlayer structure seems to be very similar to the (1×1)‐4CO structure reported by Jung et al. in this potential region, who assigned it to a high‐density phase with a full coverage of all Pt top sites by CO_ad_.4b However, we observe a distinct vertical modulation of this adlayer in form of a (2×2) superlattice, formed by significantly brighter maxima. This superstructure is difficult to rationalize by a true (1×1) adlayer of CO_ad_, where all molecules adsorb on equivalent sites and thus should have an identical apparent height. In video sequences at a constant potential of 0.20 V (Figure 2 b), we again find a highly dynamic behavior of the adlayer. In particular, the (2×2) superlattice modulation is only locally defined and can shift to one of the three other symmetrically equivalent sublattices on the Pt(111) substrate. Such shifts can occur over large portions of the imaged area on the 100 ms time scale (Figure 2 b). It is thus likely that this vertical modulation would be not (or much less) present in conventional STM images, recorded on the time scale of minutes.

At even more positive potentials (Figure 1 d), the difference in apparent brightness of the CO_ad_, that is, the vertical modulation of the (2×2) superlattice, becomes weaker. The superlattice order can decrease to an extent, where only a slight preference for local distances of 2d_Pt_ between brighter CO_ad_ rather than well defined (2×2) domains are visible. Furthermore, also the temporal fluctuations are so fast that little correlation between the brighter CO_ad_ subsequent STM images exists (Figure 2).

At potentials above 0.30 V we observe a sudden transformation to the well‐known $\left(\sqrt{19}\times \sqrt{19}\right)$ R23.4°‐13CO phase. Although this study focuses on the regime prior to this surface phase transition, we note that the transition potential and the width of the potential regime where the apparent (1×1) adlayer is observed, depends on the amount of CO in solution. With increasing CO concentration the (1×1) → $\left(\sqrt{19}\times \sqrt{19}\right)$ R23.4°‐13CO transition is shifted to more positive potentials, which is in accordance with previous studies and most probably caused by CO re‐adsorption.4b, 6a, 18 In the following, we assume that the competition of CO pre‐oxidation and CO re‐adsorption establishes a stationary potential‐dependent CO_ad_ coverage on the Pt(111) surface that is in between that of the two ordered adlayer phases (i.e., 0.68 ML≤*θ*_CO_≤0.75 ML).

As already discussed above, it is highly unlikely that the apparent (1×1) phase corresponds to a full monolayer, in which each Pt top site is occupied by a CO_ad_. However, the observations at 0.12 V point towards a more dynamic picture, where significant fluctuations in the CO_ad_ adsorption sites occur. We suggest that these site fluctuations become possible by the creation of defects in the (2×2)‐3CO adlayer via pre‐oxidation. The lower local adlayer density near these defects allows CO_ad_ in energetically unfavorable threefold‐hollow sites to move to top or near‐top sites. To rationalize the observations at more positive potentials, we recall that the STM provides a time‐averaged image of the adlayer structure, if the CO_ad_ mobility is higher than the STM's line scan rate, that is, if the CO_ad_ residence times in the sites are ≤0.1 ms, as was suggested by measurements in UHV.^11^ Because CO_ad_ in top sites appear much brighter than those in hollow sites (see Figure 1 a), the STM images will be dominated at sufficiently high defect density by the time‐averaged CO_ad_ occupancy of the top sites. Thus, the variations in CO_ad_ brightness with in the apparent (1×1) phase reflect different probabilities for the molecules to reside in these sites. The latter is equal to one for the CO_ad_(0), which occupy top sites in the defect‐free (2×2)‐3CO, but much lower for the remaining 3 top sites in the unit cell. This explains the (2×2) superperiodicity of the apparent (1×1) adlayer. With increasing defect density (i.e., increasing potential), the difference in occupancy between CO_ad_(0) and the other top sites decreases. Furthermore, the CO_ad_(0) may locally shift to one of the 3 other symmetrically equivalent (2×2) sublattices. This may be viewed as a (2×2) domain boundary fluctuation and could be directly observed at 0.12 V, where the surface dynamics is slower (Supporting Information, Figure 1S). These effects will gradually lead to an adlayer, in which the average occupancy of the Pt top sites is rather uniform on the millisecond time scale.

This scenario is supported by periodic DFT calculations, which were performed using a (6×6) surface unit cell with a (2×2)‐3CO adlayer. A defect site is then created by removing one CO molecule from either an hcp (V_hcp_) or an fcc (V_fcc_) site (see Figure 3) which is energetically less costly than creating a vacancy at a top site. According to the calculated energy changes and diffusion barriers, only the jump of a CO molecule from an adjacent three‐fold hollow site onto the vacancy site is associated with an energy gain and these jumps are also much more likely than jumps from the adjacent top sites. Therefore we keep the CO molecules at the CO_ad_(0) sites fixed in the following considerations. Three CO jumps from hcp sites toward the top sites adjacent to an fcc vacancy lead to an energetically favorable local energy minimum configuration (3CO_top1_) with a local (1×1) structure in form of a triangular CO hexamer (upper left corner of Figure 4 and Figure 3S). In this structure, all CO molecules are located at the edge and not exactly located at top sites, but shifted away from the center and slightly tilted (Figure 3S j), This reduces the mutual repulsion between the CO molecules and leads to stabilization of these hexamers, which is not possible in less compact (Figures 3S k,m) and larger local (1×1) CO structures (Figure 3S n).

**Figure 3:**
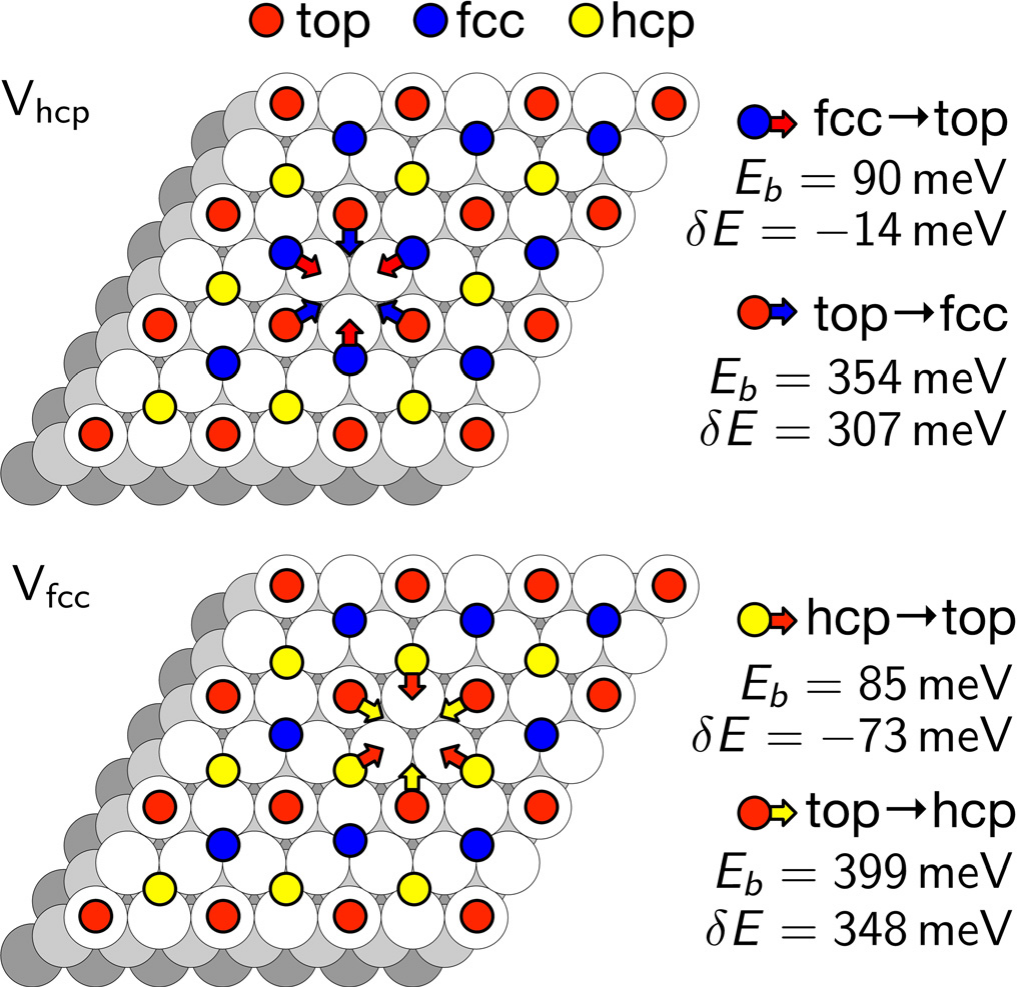
CO jumps allowed in the CO adlayer after creating a CO vacancy. E_b_ denotes the height of the diffusion barrier, δE the energy change between final and initial state of the jump.

**Figure 4:**
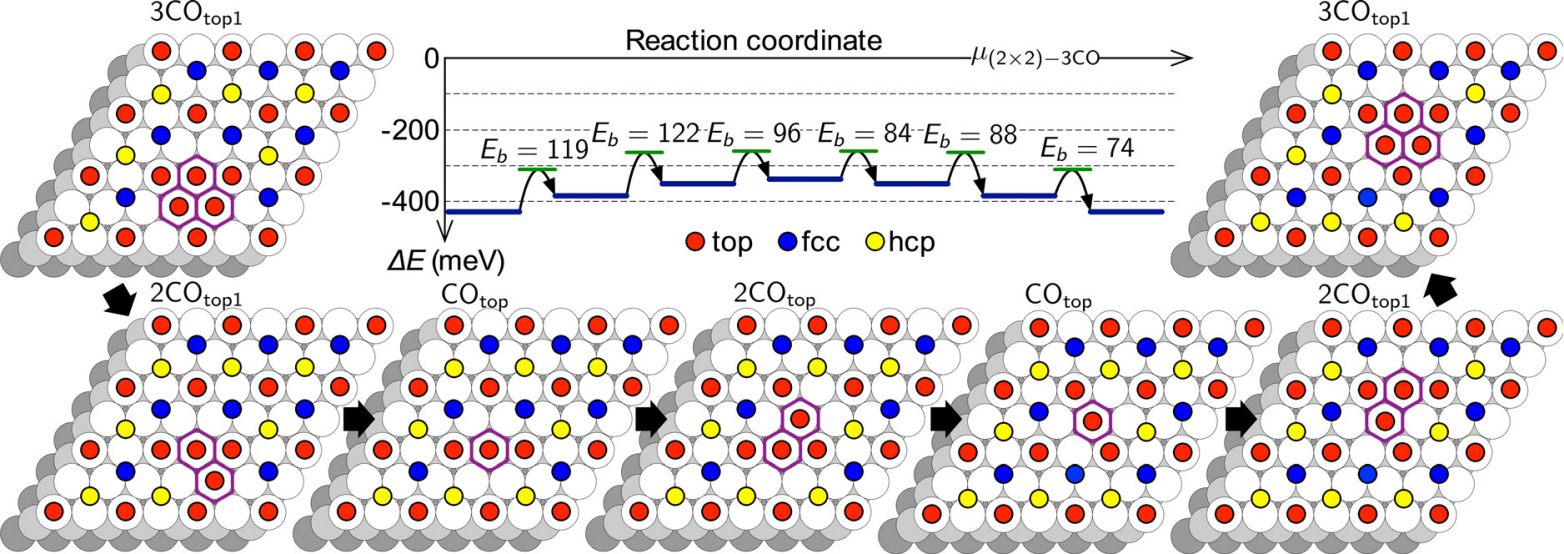
Scenario of the propagation of a local (1×1) CO hexamer to a neighboring (2×2) cell together with the calculated potential energy curve for this propagation mechanism. In addition to the CO_ad_(0), further top sites are transiently occupied by CO (indicated by purple frames) due to site jumps of individual CO.

Quasi‐collective surface diffusion of the CO hexamer is possible by rearrangement via single CO jumps, which all have relatively small diffusion barriers of less than 0.13 eV (Figure 4). By six consecutive CO jumps the local (1×1) hexamer can thus propagate to a neighboring (2×2) cell. Thus, the high mobility of single CO molecules leads also to a high mobility of local (1×1) CO structures.

This behavior resembles the “door‐opening” mechanism, proposed recently to explain the fast diffusion of O_ad_ on a fully CO‐covered Ru(0001) surface.2d, 17 Also here, local density fluctuations in the CO_ad_ adlayer intermittently create O_ad_ diffusion pathways with low activation energy. In our case, the weaker binding of CO_ad_ at certain adsorption sites of the Pt(111) surface and the possibility of density fluctuations near point defects in the (2×2)‐3CO adlayer likewise create pathways for CO_ad_ to diffuse to other adsorption sites. Because the differences in the adsorption energies of the involved sites are sufficiently low for thermal site fluctuations, long‐range transport is possible. To further verify this scenario, we are in the process of performing kinetic Monte Carlo simulations based on the DFT calculations (to be published).

This mechanism enables a high CO_ad_ surface mobility during CO pre‐oxidation, even in the presence of ordered adlayers of high packing density. It thus ensures effective mixing of CO_ad_ and OH_ad_ on the electrode surface and explains why CO electrooxidation can be described by a Langmuir‐Hinshelwood type reaction within the “mean‐field approximation”.10b, 12, 19 Similar process may occur in other electrocatalytic systems, where high surface coverages of adsorbates are the rule rather than the exemption.

## Conflict of interest

The authors declare no conflict of interest.

## Supporting information

As a service to our authors and readers, this journal provides supporting information supplied by the authors. Such materials are peer reviewed and may be re‐organized for online delivery, but are not copy‐edited or typeset. Technical support issues arising from supporting information (other than missing files) should be addressed to the authors.

Supplementary

Supplementary
