# Supplementary material for: The Dynamic Nature of CO Adlayers on Pt(111) Electrodes
Source: Angew Chem Int Ed Engl. 2020 Feb 18;59(15):6182–6. doi: 10.1002/anie.201913412 (PMC7187359; doi:10.1002/anie.201913412)
Supplement: Supplementary file 1 — Supplementary [file ANIE-59-6182-s001.pdf]

Angewandte Chemie
Eine Zeitschrift der Gesellschaft Deutscher Chemiker

Supporting Information

The Dynamic Nature of CO Adlayers on Pt(111) Electrodes

Jie Wei, Reihaneh Amirbeigiarab, Yan-Xia Chen, Sung Sakong, Axel Gross, and Olaf M. Magnussen**

anie_201913412_sm_miscellaneous_information.pdf
anie_201913412_sm_Movie_0.3V.mp4

Experimental Section

Electrochemical procedures

The experiments were performed at controlled potential at room temperature (≈298 K), using a homebuilt video-STM for *in situ* measurements in electrochemical environment.[1] The STM data were recorded in constant height mode at 10 images/s, using polypropylene-covered tungsten tips. Prior to the measurements, the Pt(111) electrode was annealed in an induction oven for 30 min at ≈1000°C in Ar containing 2% CO and then cooled down to room temperature in the same atmosphere. 0.1 M H_2SO_4 saturated with CO was used as electrolyte solution, prepared from ultrapure H_2SO_4 (Merck) and ultrapure water and saturated by bubbling for 10 min with pure CO gas (Linde, minican). Potentials were measured versus a Ag/AgCl (KCl sat.) reference electrode.

Density functional theory calculations

Periodic DFT calculations were performed using the software package VASP.[2] The exchange-correlation energies are evaluated as suggested by Hammer and Nørskov, known as a revised version of the Perdew-Burke-Ernzerhof (RPBE) functional.[3] The Pt(111) surface was modeled by a five-layer slab with a (6x6) surface unit cell. The dispersion interactions between adsorbate-adsorbate and adsorbate-first Pt layer of the slab are included by the semi-empirical D3 dispersion correction scheme of Grimme.[4] The RPBE-D3 approach has recently been shown to give a reliable description of the site preference within the CO/Pt(111) system.[5] Diffusion barriers of CO are determined by the nudged elastic band (NEB) method with four images.[6] The wave functions are expanded up to 400 eV using a plane-wave basis set, and the integral over the first Brillouin zone is done on a 3x3x1 k-point mesh.

Supplementary STM data

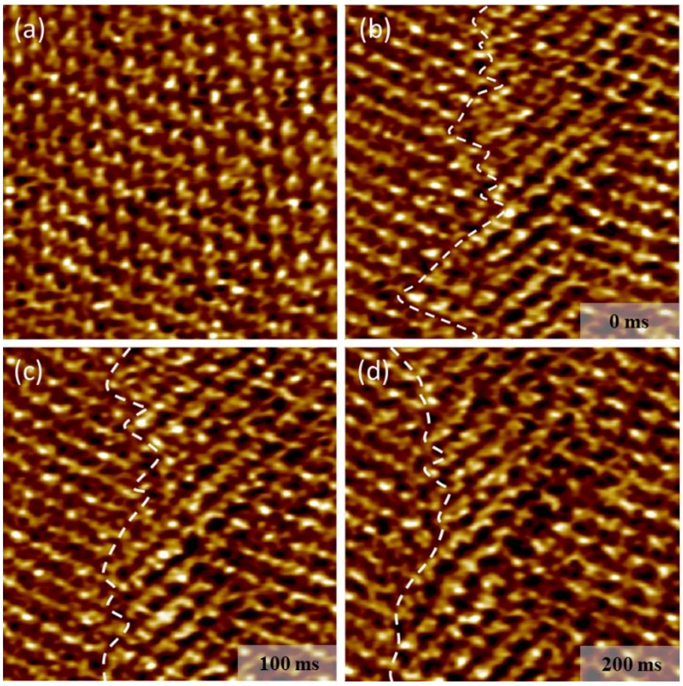

Figure 1S. Sequence of *in situ* video STM images (7 nm × 7 nm) of Pt(111) in CO-saturated 0.1 M H_2SO_4 solution at 0.12 V (7 nm × 7 nm), showing fast positional fluctuations of a domain boundary within the (2 × 2)-3CO adlayer

0.3V.mp4: Video STM sequence of CO/Pt(111) (8 nm × 8 nm), recorded at 0.30 V at 10 Hz.

Supplementary DFT results

The CO molecules occupy a high symmetry site on Pt(111) surface when all six neighboring high symmetry sites are free. We have tested the coverage dependence on the electrode potential, as shown in Figure 2S a. The computational electrode is used to determine the formation enthalpy with respect to the gas-phase CO_2 molecule. The fully covered CO only exists below -0.36 V, i.e., in the hydrogen evolution regime. The (2x2)-3CO (Figure 2S b) is stable in the range between -0.36 and 0.20 V. Vacancy formation starts above 0.2 V. We find the DFT calculations to be consistent with the STM experiments.

Any CO jump in a complete (2x2)-3CO adlayer leads to a CO pair placed at the neighboring high symmetry sites. Thus, the (2x2)-3CO adlayer is rigid, and a CO fluctuation is not expected below 0.2 V. When a CO vacancy starts to be formed above 0.2 V, the fluctuating nature of the CO adlayer becomes significant. In Figure 3S, we illustrate the considered CO configurations after CO vacancy formation together with their formation energies. The local minimum energy configuration $3CO_{top1}$ is shown in Figure 3S j.

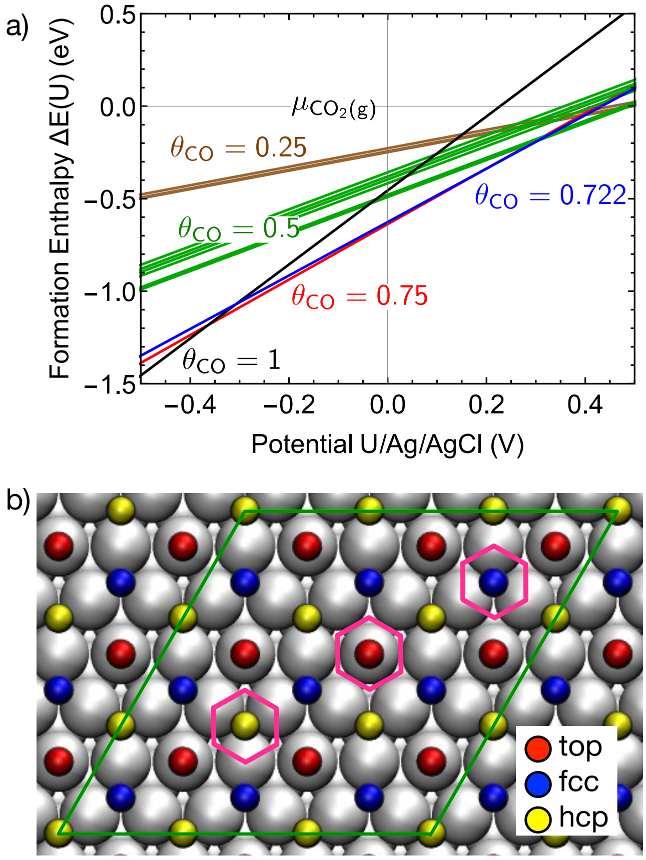

Figure 2S. (a) The potential dependence of CO coverage on Pt(111) electrode. (b) Atomic configuration of the (2x2)-3CO structure on Pt(111).

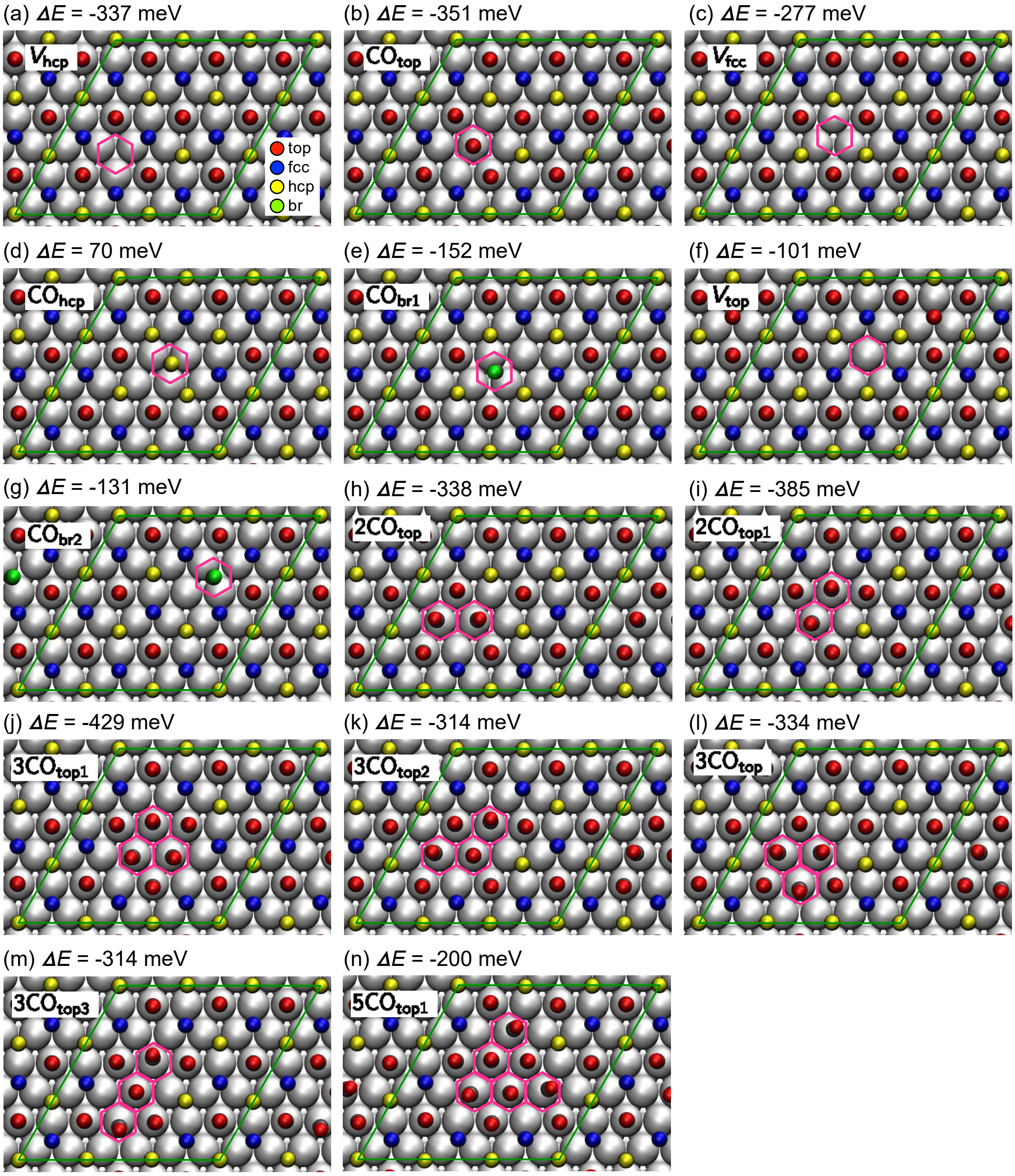

Figure 3S. Disordered CO adlayer configurations after creating a CO vacancy. The energies refer to the complete (2x2)-3CO configuration, i.e., $\mu_{CO} = \mu$[(2x2)-3CO].

References

[1] O. M. Magnussen, *Chemistry–A European Journal* **2019**.
[2] G. Kresse, J. Furthmüller, *Phys Rev B* **1996**, *54*, 11169.
[3] B. Hammer, L. B. Hansen, J. K. Nørskov, *Phys Rev B* **1999**, *59*, 7413.
[4] S. Grimme, J. Antony, S. Ehrlich, H. Krieg, *The Journal of chemical physics* **2010**, *132*, 154104.
[5] D. Mahlberg, S. Sakong, K. Forster-Tonigold, A. Groß, *Journal of chemical theory and computation* **2019**, *15*, 3250-3259.
[6] G. Henkelman, B. P. Uberuaga, H. Jónsson, *The Journal of chemical physics* **2000**, *113*, 9901-9904.
